# Early Outcomes With Cerebral Embolic Protection During Transcatheter Aortic Valve Replacement in Patients With Atrial Fibrillation

**DOI:** 10.1016/j.shj.2024.100353

**Published:** 2024-09-02

**Authors:** Shashank Shekhar, Amar Krishnaswamy, Grant Reed, James Yun, Rishi Puri, Samir Kapadia

**Affiliations:** Department of Cardiovascular Medicine, Heart, Vascular and Thoracic Institute, Cleveland Clinic, Cleveland, Ohio

**Keywords:** Cerebral embolic protection, Mortality, Sentinel, Stroke, Transcatheter aortic valve replacement

## Abstract

**Background:**

Limited studies are available which aim to identify patient populations that would potentially benefit from the use of cerebral embolic protection devices (CPDs) during transcatheter aortic valve replacement (TAVR). We aimed to analyze the impact of CPD use during TAVR among patients with atrial fibrillation (AF).

**Methods:**

Data on adult TAVR patients with a concomitant diagnosis of AF was obtained from the 2017-2020 National Readmissions Database. Stroke, major stroke, in-hospital mortality, and 30-day readmission rates were compared between the CPD and no-CPD cohorts in a propensity score matched analysis. Association of CPD use with adverse events was analyzed using multivariable logistic regression models.

**Results:**

Of 100,928 eligible TAVR patients with AF, CPD was used in 6.9% of patients with a mean age of 80 years. CPD use was independently associated with lower overall stroke (1.7% vs. 2.2%; odds ratio [OR] 0.81 [95% CI 0.68-0.98]; *p* = 0.032), major stroke (1.2% vs. 1.8%; OR 0.69 [0.55-0.86]; *p* = 0.001), in-hospital mortality (0.9 vs. 1.5%; OR 0.56 [0.43-0.72]; *p* < 0.001), and lower 30-day readmission rates (12.7% vs. 14.7%; OR 0.87 [0.81-0.94]; *p* < 0.001). Reduction in adverse events with CPD was noted in high-volume but not in low-volume TAVR centers.

**Conclusions:**

The present point towards clear benefits of CPD use among patients with AF undergoing TAVR. In anatomically eligible patients, the potential benefit of debris capture may be considered especially as younger and lower risk patients become eligible for TAVR. Data from future trials and registries are required to further corroborate our findings.

## Highlights

• Stroke remains a formidable cause of morbidity, mortality, and resource utilization in transcatheter aortic valve replacement (TAVR) patients.
• Sentinel cerebral embolic protection device (CPD) is designed to capture and remove debris thereby reducing the risk of stroke.
• There is an interest in identifying patients who may benefit from the use of the CPD.
• Patients with atrial fibrillation undergoing TAVR with CPD had lower overall stroke, and major stroke.
• Thirty-day all cause readmissions were lower in patients with atrial fibrillation undergoing TAVR with CPD use.
• Future studies from other registries and clinical trials are required to corroborate our findings and to identify candidates for CPD use.

## Introduction

Stroke remains a devastating and feared complication of the revolutionary transcatheter aortic valve replacement (TAVR) procedure, affecting nearly 2% of TAVR patients.^1^^,^^2^ Post-TAVR stroke is associated with increased mortality.^3^^,^^4^ In order to decrease the potential complication of stroke relating to the TAVR procedure, cerebral embolic protection devices (CPDs) have been developed.^5^

The SENTINEL CPD (Boston Scientific Corp, Boston, Massachusetts) was approved by the United States Food and Drug Administration for stroke prevention during TAVR^6^ following results from the SENTINEL randomized controlled trial (RCT) which demonstrated the safety of the device in 363 high-risk patients with aortic stenosis (AS).^7^^,^^8^ The recently published results of the PROTECTED TAVR RCT (Stroke PROTECTion With SEntinel During Transcatheter Aortic Valve Replacement) further demonstrated the efficacy of the CPD device in reducing disabling stroke among 3000 TAVR patients across 50 global sites, at all surgical levels (0.5% in protected vs. 1.3% in unprotected, *p* = 0.02).^6^ However, the RCT did not aim to identify patients with specific underlying comorbidities who would benefit from CPD use.

Patients with atrial fibrillation (AF) are known to be at a high-risk for stroke, possibly due to cardioembolic events in the left atrial appendage.^3^ In an effort to identify candidates who may potentially benefit from CPD, we aimed to assess the outcomes of patients with AF who underwent TAVR with and without the use of CPD.

## Methods

### Data Source

The present study was conducted from data obtained from Nationwide Readmissions Database (NRD) (2017-2020) maintained by the Healthcare Cost and Utilization Project (HCUP) of the Agency for Healthcare Research and Quality in the United States.^9^ The data set is the largest publicly available database representing ∼52% of all US hospitalizations. Patients are tracked using verifiable linkage numbers during a calendar year, i.e., from January to the end of December. Patient characteristics, diagnoses, and procedures are coded in the database using the International Classification of Diseases (ICD) ninth and 10th Revision Clinical Modification and Procedure Coding System codes. Since the data are publicly available and contain deidentified information, approval from the institutional review board was not required for this study. The Strengthening the Reporting of Observational Studies in Epidemiology (STROBE) checklist was used when writing the present report.^10^

#### Study Design

Supplementary Table 1 lists the ICD-10 Clinical Modification and Procedure Coding System codes used for the present study. Data on adult transfemoral TAVR recipients were extracted using verified ICD-10 codes (‘02RF37Z,’ ‘02RF38Z,’ ‘02RF3JZ,’ and ‘02RF3KZ’).^11^ Patients with concomitant surgery (surgical aortic valve replacement, coronary bypass surgery, or thoracic aorta surgery) were excluded, and only those with a diagnosis of AF were included in the analysis. Hospitals were categorized into metropolitan teaching hospitals vs others.

### Definitions and Outcomes

The variable *DISPUNIFORM* provided by the HCUP contains information on the discharge disposition of patients and is coded from ‘1 to 7’ and ’20,’ corresponding to routine home/self-care discharge, ‘20’ representing death, and the rest representing discharge to another hospital/nursing facility (short-term hospital, skilled nursing facility, intermediate care facility, another type of facility, or home health care). This information was used to define “major stroke” in the present study as stroke which resulted in death or discharge to another hospital/nursing facility.

The variable *HOSP_NRD* provided by the HCUP was used for the calculation of annual hospital procedural volumes. Hospitals were categorized into low-volume TAVR centers if the number of procedures performed was ≤99 TAVR procedures in each year, and high-volume TAVR centers if the number of procedures performed was ≥100 TAVR procedures.

Overall stroke, major stroke, length of stay, routine discharges, in-hospital mortality, and 30-day readmission outcomes were compared between patients who underwent TAVR with and without the use of a CPD (‘X2A5312’). The weight variable provided by the HCUP, *DISCWT*, was used in order to obtain national estimates.

### Statistical Analysis

We compared patient characteristics and outcomes between the CPD and no-CPD cohorts using Fisher exact text or chi-square test for categorical variables, and Mann-Whitney *U* test or Student *t* test continuous variables. To mitigate the risk of confounding and selection bias, a propensity score matching model was developed using logistic regression to derive 2 matched groups. The MatchIt program in R statistical software was used to perform a 1:2 nearest neighbor matching, using a caliper of 0.1. Details of the propensity matching appear in the Supplementary Materials (Figures S1 and S2). All variables were also included in a multivariable logistic regression model in order to analyze the impact of CPD on overall stroke, major stroke, in-hospital mortality, and 30-day readmission rates among patients with AF undergoing TAVR. In the present study, a 2-sided *p* value of <0.05 was considered statistically significant. Data extraction and statistical analyses were performed using SAS (SAS Institute Inc, Cary, North Carolina), SPSS Statistics version 29 (IBM SPSS, Armonk, New York), and RStudio.

## Results

### Study Cohort

**Figure 1:**
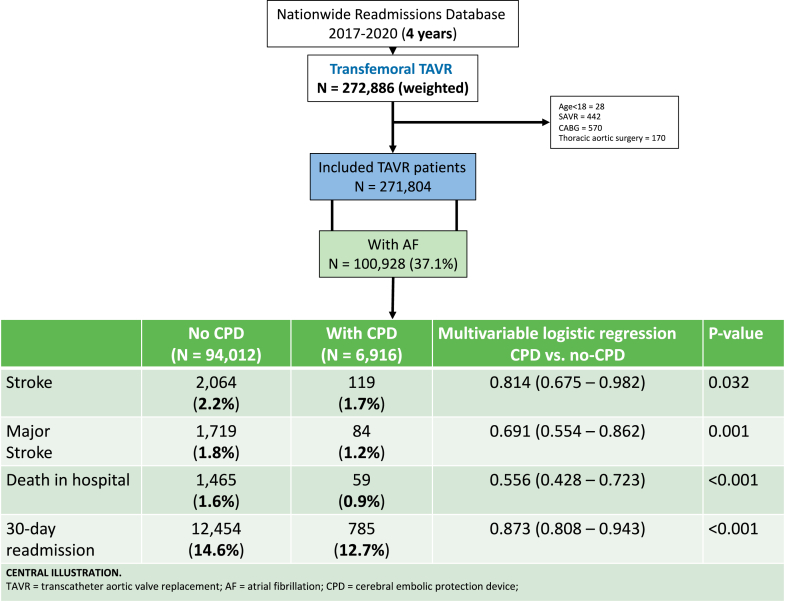

Figure 1 depicts the patient selection for the present study. Of 271,804 eligible transfemoral TAVR patients, 100,928 (37.1%) patients had an underlying diagnosis of AF and were included in the final analysis. The overall mean (SD) age of patients was 80.4 (7.7) years, with the majority being males (58.5%), being treated at metropolitan teaching hospitals (88.2%) (Table 1). CPD was used in 6916 (6.9%) of patients with a mean age of 80 years. CPD vs no-CPD patients were more commonly males (62.6% vs. 58.2%), with a lesser burden of comorbidities such as diabetes with chronic complications (14.3% vs. 17.4%), chronic pulmonary disease (24.9% vs. 26.3%), carotid artery disease (5.6% vs. 6.3%), end-stage renal disease on dialysis (2.9% vs. 4.0%), and prior coronary bypass surgery (15.6% vs. 17.7%).

**Table 1:** Baseline characteristics of TF-TAVR patients with atrial fibrillation

Characteristics	Unmatched				PS-matched		
	Overall N = 100,928	No CPD N = 94,012 (93.1%)	CPD N = 6916 (6.9%)	*p* value	No CPD N = 15,826	CPD N = 6916	*p* value
Mean age in y (SD)	80.42 (7.69)	80.44 (7.69)	80.19 (7.79)	0.255	80.36 (7.63)	80.19 (7.79)	0.418
Females	41,873 (41.5)	39,289 (41.8)	2584 (37.4)	<0.001	5974 (37.7)	2584 (37.4)	0.581
Bicuspid aortic valve	1190 (1.2)	1032 (1.1)	158 (2.3)	<0.001	358 (2.3)	158 (2.3)	0.918
Prior valve surgery	5278 (5.2)	4958 (5.3)	320 (4.6)	0.020	654 (4.1)	320 (4.6)	0.090
Metropolitan teaching hospital	88,971 (88.2)	82,467 (87.7)	6504 (94.0)	<0.001	14,933 (34.4)	6504 (94.0)	0.348
Hypertension	92,376 (91.5)	86,038 (91.5)	6338 (91.6)	0.720	14,434 (91.2)	6338 (91.6)	0.280
Diabetes without chronic complications	12,789 (12.7)	11,868 (12.6)	921 (13.3)	0.095	2141 (13.5)	921 (13.3)	0.665
Diabetes with chronic complications	17,347 (17.2)	16,359 (17.4)	988 (14.3)	<0.001	2269 (14.3)	988 (14.3)	0.914
Chronic pulmonary disease	26,450 (26.2)	24,731 (26.3)	1719 (24.9)	0.008	3782 (23.9)	1719 (24.9)	0.123
Carotid artery disease	6344 (6.3)	5959 (6.3)	385 (5.6)	0.011	783 (4.9)	385 (5.6)	0.052
ESRD on dialysis	3921 (3.9)	3721 (4.0)	200 (2.9)	<0.001	439 (2.8)	200 (2.9)	0.622
Prior CABG	17,699 (17.5)	16,620 (17.7)	1079 (15.6)	<0.001	2354 (14.9)	1079 (15.6)	0.159
Prior PCI	21,338 (21.1)	19,825 (21.1)	1513 (21.9)	0.212	3438 (21.7)	1513 (21.9)	0.799
Prior PPM	15,422 (15.3)	14,341 (15.3)	1081 (15.6)	0.402	2322 (14.7)	1081 (15.6)	0.062
Prior CVA	600 (0.6)	560 (0.6)	40 (0.6)	0.857	70 (0.4)	40 (0.6)	0.174

*Notes*. Results presented as N (%) unless otherwise mentioned.

Abbreviations: CABG, coronary artery bypass grafting; CPD, cerebral protection device; CVA, cerebrovascular accident; ESRD, end-stage renal disease; PCI, percutaneous coronary intervention; PPM, pacemaker; PS, propensity score; TAVR, transcatheter aortic valve replacement; TF, transfemoral.

## Outcomes

Table 2 shows the overall outcomes of patients as well as outcomes of patients stratified by the use of CPD. Around 2.2% of all TAVR patients suffered a stroke in hospital, with 1.8% suffering a major stroke, and 1.5% in-hospital deaths. The median (interquartile range) length of stay for patients was 2 (1-5) days, and 64% of patients were routinely discharged to home. The overall 30-day readmission rate was 14.5% among TAVR patients with AF.

**Table 2:** Outcomes of TF-TAVR patients with atrial fibrillation

Outcomes	Overall N = 100,928	Unmatched			PS-matched		
		No CPD N = 94,012 (93.1%)	CPD N = 6916 (6.9%)	*p* value	No CPD N = 15,826	CPD N = 6916	*p* value
Outcomes of patients							
Overall stroke	2183 (2.2)	2064 (2.2)	119 (1.7)	0.009	344 (2.2)	119 (1.7)	0.026
Major stroke∗	1803 (1.8)	1719 (1.8)	84 (1.2)	<0.001	287 (1.8)	84 (1.2)	0.001
Median length of stay in d (IQR)	2.0 (1-5)	2.0 (1-5)	2.0 (1-4)	<0.001	2.0 (1-5)	2 (1-4)	<0.001
Routine discharge	64,806 (64.2)	60,094 (63.9)	4712 (68.1)	<0.001	10,398 (65.7)	4712 (68.1)	<0.001
In-hospital mortality	1524 (1.5)	1465 (1.6)	59 (0.9)	<0.001	243 (1.5)	59 (0.9)	<0.001
30-d readmission rate†	13,239/91,192 (14.5)	12,454/85,025 (14.6)	785/6167 (12.7)	<0.001	2101/14,325 (14.7)	785/6167 (12.7)	<0.001

Multivariable logistic regression (CPD vs. no CPD)			
	Odds ratio	95% CI	*p* value
Overall stroke	0.814	0.675-0.982	0.032
Major stroke∗	0.691	0.554-0.862	0.001
In-hospital mortality	0.556	0.428-0.723	<0.001
30-d readmission rate†	0.873	0.808-0.943	<0.001

*Notes*. Results presented as N (%) unless otherwise mentioned.

Abbreviations: CPD, cerebral protection device; IQR, interquartile range; PS, propensity score; TAVR, transcatheter aortic valve replacement; TF, transfemoral.

∗ Major stroke was defined as stroke leading to death or nonroutine discharge.

† 30-d readmissions were calculated among patients discharged alive during the first 11 mo of the y to allow for 30-d follow-up.

In the unmatched analysis, those who underwent TAVR with vs without CPD had lower overall stroke (1.7% vs. 2.2%; *p* = 0.009), major stroke (1.2% vs. 1.8%; *p* < 0.001), in-hospital mortality (0.9% vs. 1.6%; *p* < 0.001), shorter length of stay (2 [1-4] vs. 2 [1-5] days; *p* < 0.001), higher routine discharges (68.1% vs. 63.9%; *p* < 0.001), and lower 30-day readmission rates (12.7% vs. 14.6%; *p* < 0.001). Similarly, in the propensity score-matched analysis, CPD patients had lower overall stroke (1.7% vs. 2.2%; *p* = 0.026), major stroke (1.2% vs. 1.8%; *p* = 0.001), in-hospital mortality (0.9% vs. 1.5%; *p* < 0.001), shorter length of stay (2 [1-4] vs. 2 [1-5] days; *p* < 0.001), higher routine discharges (68.1% vs. 65.7%; *p* < 0.001), and lesser readmission rates (12.7% vs. 14.7%; *p* < 0.001). The multivariable logistic regression model showed that CPD use was independently associated with lower overall stroke (odds ratio, 0.81 [95% CI 0.68-0.98]; *p* = 0.032), major stroke (0.69 [0.55-0.86]; *p* = 0.001), in-hospital mortality (0.56 [0.43-0.72]; *p* < 0.001), and lower 30-day readmission rates (0.87 [0.81-0.94]; *p* < 0.001.

### Outcomes Stratified by Annual Hospital Procedural Volume

Of 100,928 included patients, 36,619 (36.3%) underwent TAVR at low-volume centers (3.1% with CPD; 96.9% without CPD), while 64,309 (63.7%) underwent TAVR at high-volume centers (91.0% without CPD; 9.0% with CPD) (Table 3). In the low-volume TAVR centers, although CPD patients had higher routine discharges (70.2% vs. 66.9%; *p* = 0.018), there was no significant difference in overall stroke (*p* = 0.078), major stroke (*p* = 0.120), in-hospital mortality (*p* = 0.412), or the 30-day readmission rates (*p* = 0.899). In the high-volume TAVR centers, CPD patients had lower overall stroke (1.8% vs. 2.2%; *p* = 0.032), major stroke (1.2% vs. 1.9%; *p* < 0.001), in-hospital mortality (0.7% vs. 1.5%; *p* < 0.001), shorter length of stay (2 [1-4] vs. 2 [1-5] days; *p* < 0.001), higher routine discharges (67.7% vs. 62.1%; *p* < 0.001), and lesser 30-day readmission rates (12.2% vs. 14.4%; *p* < 0.001).

**Table 3:** Outcomes of TF-TAVR patients with atrial fibrillation stratified by annual hospital procedural volume

Outcomes	Low-volume TAVR center (≤99 procedures/y)				High-volume TAVR center (≥100 procedures/y)			
	Overall N = 36,619	No CPD N = 35,474	CPD N = 1145	*p* value	Overall N = 64,309	No CPD N = 58,538	CPD N = 5771	*p* value
Overall stroke	783 (2.1)	767 (2.2)	16 (1.4)	0.078	1400 (2.2)	1297 (2.2)	103 (1.8)	0.032
Major stroke	631 (1.7)	618 (1.7)	13 (1.1)	0.120	1172 (1.8)	1101 (1.9)	71 (1.2)	<0.001
Median length of stay in d (IQR)	2 (1-4)	2 (1-4)	2 (1-4)	<0.001	2 (1-5)	2 (1-5)	2 (1-4)	<0.001
Routine discharge	24,533 (67.0)	23,729 (66.9)	804 (70.2)	0.018	40,273 (62.6)	36,365 (62.1)	3908 (67.7)	<0.001
In-hospital mortality	625 (1.7)	609 (1.7)	16 (1.4)	0.412	899 (1.4)	856 (1.5)	43 (0.7)	<0.001
30-d readmissions	4949/32,879 (15.1)	4796/31,872 (15.0)	153/1007 (15.2)	0.899	8290/58,313 (14.2)	7658/53,153 (14.4)	632/5160 (12.2)	<0.001

*Notes*. Unweighted numbers were used for the calculation and reporting of outcomes by annual hospital procedural volume.

Abbreviations: CPD, cerebral protection device; IQR, interquartile range; TAVR, transcatheter aortic valve replacement; TF, transfemoral.

## Discussion

In the present study from a nationally representative all-comers database, we found that CPD use among patients with AF undergoing TAVR was associated with lower rates of overall stroke, major stroke, in-hospital mortality, and consequently shorter duration of hospital stay, more frequent routine discharges, and lower-30-day readmission rates. Furthermore, CPD use in high-volume TAVR centers showed beneficial effects in reducing adverse events, although this reduction in adverse events was not noticed among patients undergoing TAVR at low-volume TAVR centers.

During valve deployment, calcium deposits get dislodged into the carotid or the vertebral arteries leading to stroke. The Sentinel CPD consists of 2 filters, one which is threaded into the right radial artery, and the second which is deployed to the carotid artery, with a logical aim to capture the dislodged debris. The CPD protects only ∼80% of cerebral circulation, which is perhaps why the largest CPD RCT, PROTECTED TAVR, suggested benefits of CPD use in preventing disabling stroke, but not overall stroke.^6^ Limited studies have been performed to examine the effects of the CPD device among select patients. In a study from the National Inpatient sample, Zhang et al. reported lower overall stroke risk as well as lower composite safety endpoint of in-hospital death and stroke with CPD among patients with bicuspid aortic valve undergoing TAVR.^11^ Recently, our group examined the effects of CPD in TAVR patients with a failed bioprosthesis and found that CPD use was associated with lower adverse events including major stroke in this important patient population.^12^ With the Sentinel device being in its seventh year of use after the United States Food and Drug Administration approval in 2017, there is an interest in recognizing high stroke-risk patients who may potentially benefit from CPD use, given the lack of conclusive RCT data depicting risk reduction among all-comers.

Patient- and procedure-related factors such as prior history of stroke, AF, severe calcification, prolonged procedure time, rapid pacing, and extensive aortic manipulation have been previously described as predictors of stroke following TAVR.^3^ Studies have shown potential benefits of CPD use in high-risk patients.^6^^,^^13^ A recent meta-analysis of 6 RCTs reported that the Sentinel CPD was safe and reduced disabling stroke during the TAVR procedure in select patients at high to intermediate surgical risk.^3^ A reduction in imaging markers for stroke after TAVR has been reported in prior analyses of RCTs.^14^, ^15^, ^16^ Patients with AF are considered high-risk, with more advanced cardiovascular disease. Studies have shown AF to be an independent predictor of worse outcomes in patients with AS.^17^^,^^18^ In a study including 1847 patients with severe AS, presence of AF was shown to be associated with poor prognosis.^19^ Furthermore, prior literature has shown AF to be a major predictor of mortality in patients with AS.^17^ In a global, patient-level analysis, Vlastra et al.^20^ reported a 5-fold higher rate of AF in TAVR patients who developed stroke. In this regard, we found that CPD was associated with lower overall stroke, major stroke, in-hospital mortality, and 30-day readmissions among patients with AF undergoing TAVR.

We also found that CPD when used in low-volume TAVR centers was not associated with a reduction in adverse events, including 30-day mortality. On the other hand, high-volume TAVR centers showed a significant reduction in overall stroke rate, major stroke rate, mortality rate, along with a shorter length of hospital stay, and more frequent routine discharges and lower 30-day readmission rate when the CPD device was used. A study from the 2018 NRD showed an inverse relation between increasing CPD volume use and in-hospital stroke.^21^ This highlights that operator experience likely plays an important role in the appropriate utilization of the CPD device, and the subsequently observed reduced adverse events. However, there could be other procedural factors such as, access site, predilation and postdilation, valve types, height of implantation, anesthesia, and others, which may also affect these observed changes.^22^, ^23^, ^24^

### Study Limitations

The present retrospective study is inherently limited by the use of an administrative database which relies on codes for capturing diagnoses and procedures. Although we adjusted for all patient characteristics to look at the association of CPD with adverse events, association does not necessarily imply causation, and results may still be driven by other unadjusted or adjusted confounders. The data source also lacks important data on surgical risk scores and neurological assessment of stroke. Procedural details such as predilation and postdilation, use of anticoagulation, medication details, anesthesia, valve types, and others are not obtainable from the data source used for the present study.

## Conclusion

The present study from an all-comers nationally representative database points toward clear benefits of CPD use among patients with AF undergoing TAVR. In anatomically eligible patients, the potential benefit of debris capture may be considered especially as younger and lower risk patients become eligible for TAVR.^25^ Data from future trials and registries are required to further corroborate our findings.

## Ethics Statement

The data utilized for the present study is derived from a publicly available database containing deidentified information. Approval from the Institutional Review Board was not required for the study.

## Funding

This study was made possible by a generous gift from Jennifer and Robert McNeil. The funders had no role in the design and conduct of the study, in the collection, analysis, and interpretation of the data, and in the preparation, review, or approval of the manuscript.

## Disclosure Statement

The authors report no conflict of interest.
