# Supplementary material for: Early Outcomes With Cerebral Embolic Protection During Transcatheter Aortic Valve Replacement in Patients With Atrial Fibrillation
Source: Struct Heart. 2024 Sep 2;9(1):100353. doi: 10.1016/j.shj.2024.100353 (PMC11864126; doi:10.1016/j.shj.2024.100353)

**Supplementary Material**

**eTable 1.** International Classification of Diseases 10^th^ Revision Clinical Modification and Procedure Coding System Codes

| **Diagnosis or procedure** | **Codes** |
| --- | --- |
| Transfemoral TAVR | 02RF37Z, 02RF38Z, 02RF3JZ, 02RF3KZ |
| Sentinel cerebral embolic protection device | X2A5312 |
| Stroke | I60, I61, I63, I9781, I9782, |
| SAVR | 02RF0, 02RF4, X2RF032, X2RF432 |
| CABG | 0210, 0211, 0212, 0213 |
| Transthoracic vessel surgery | 02RW07Z, 02RW08Z, 02RW0JZ, 02RW0KZ, 02RX07Z, 02RX0JZ, 02RX0KZ, 02RX0KZ, |
| Bicuspid aortic valve | Q230, Q231 |
| Prior valve surgery | Z952, Z953, Z953, Z954 |
| Hypertension | Codes for Elixhauser comorbidity index provided by the HCUP |
| Diabetes without chronic complications | Codes for Elixhauser comorbidity index provided by the HCUP |
| Diabetes with chronic complications | Codes for Elixhauser comorbidity index provided by the HCUP |
| Chronic pulmonary disease | Codes for Elixhauser comorbidity index provided by the HCUP |
| Atrial fibrillation | I480, I481, I482, I4891 |
| Carotid artery disease | I652 |
| ESRD requiring dialysis | N186 |
| Prior CABG | Z951, I25700, I25708, I25709, I2571, I2572, I2573, I2576, I2579, I25810, I25812, |
| Prior PCI | Z9861, Z955 |
| Prior Pacemaker | Z950 |
| Prior CVA | Z8674, I69910, I69911, I69913, I69914, I69915, I69918, I69919, |
| TAVR = transcatheter aortic valve replacement; SAVR = surgical aortic valve replacement; CABG = coronary artery bypass grafting; ESRD = end stage renal disease; PCI = percutaneous coronary intervention; PPM = pacemaker; CVA = cerebrovascular accidents; HCUP = Healthcare Cost and Utilization Project | |

**Figure S1.** Covariate Balance Plot
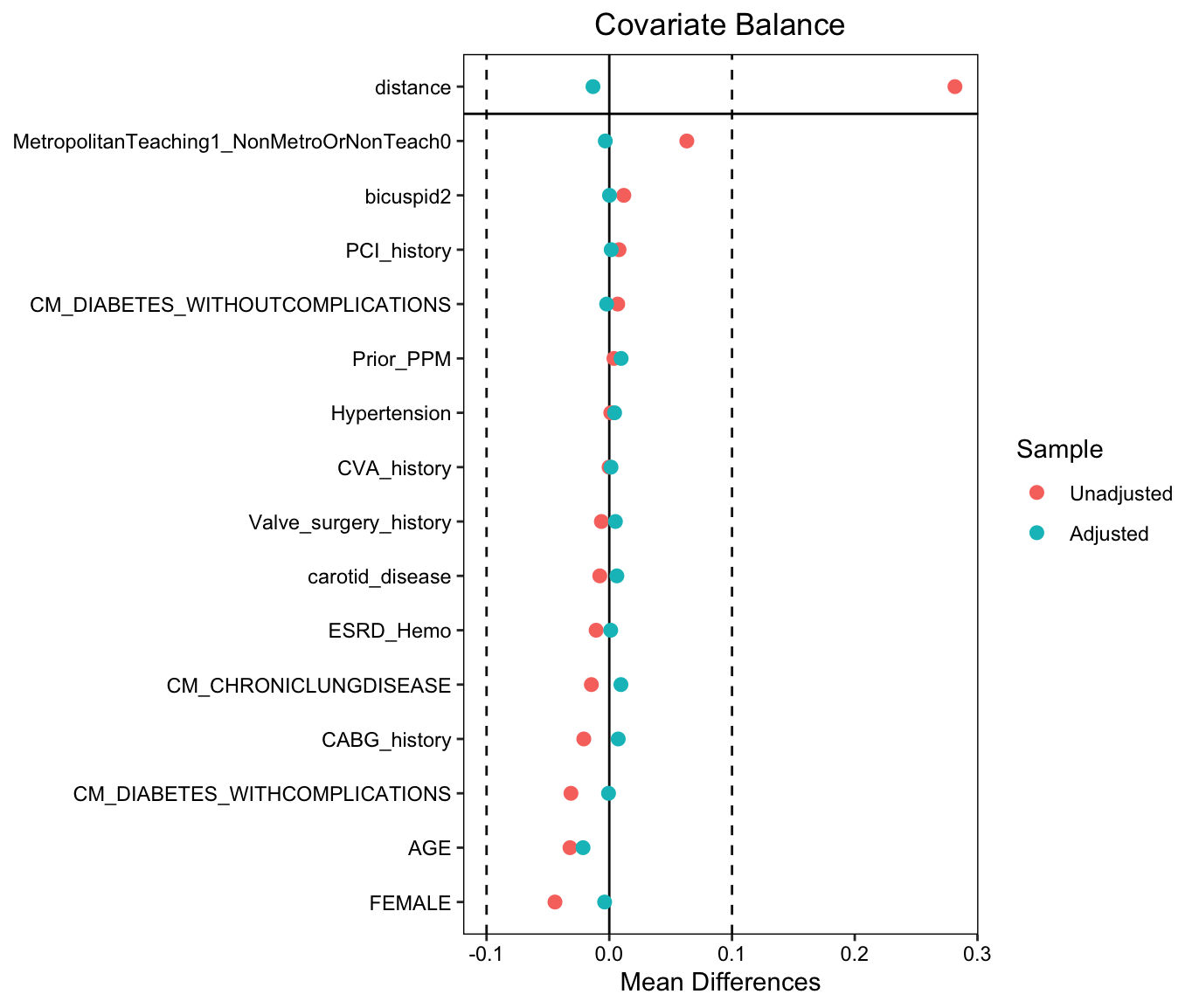


**Figure S2.** Distribution of Propensity Scores (jitter plot)


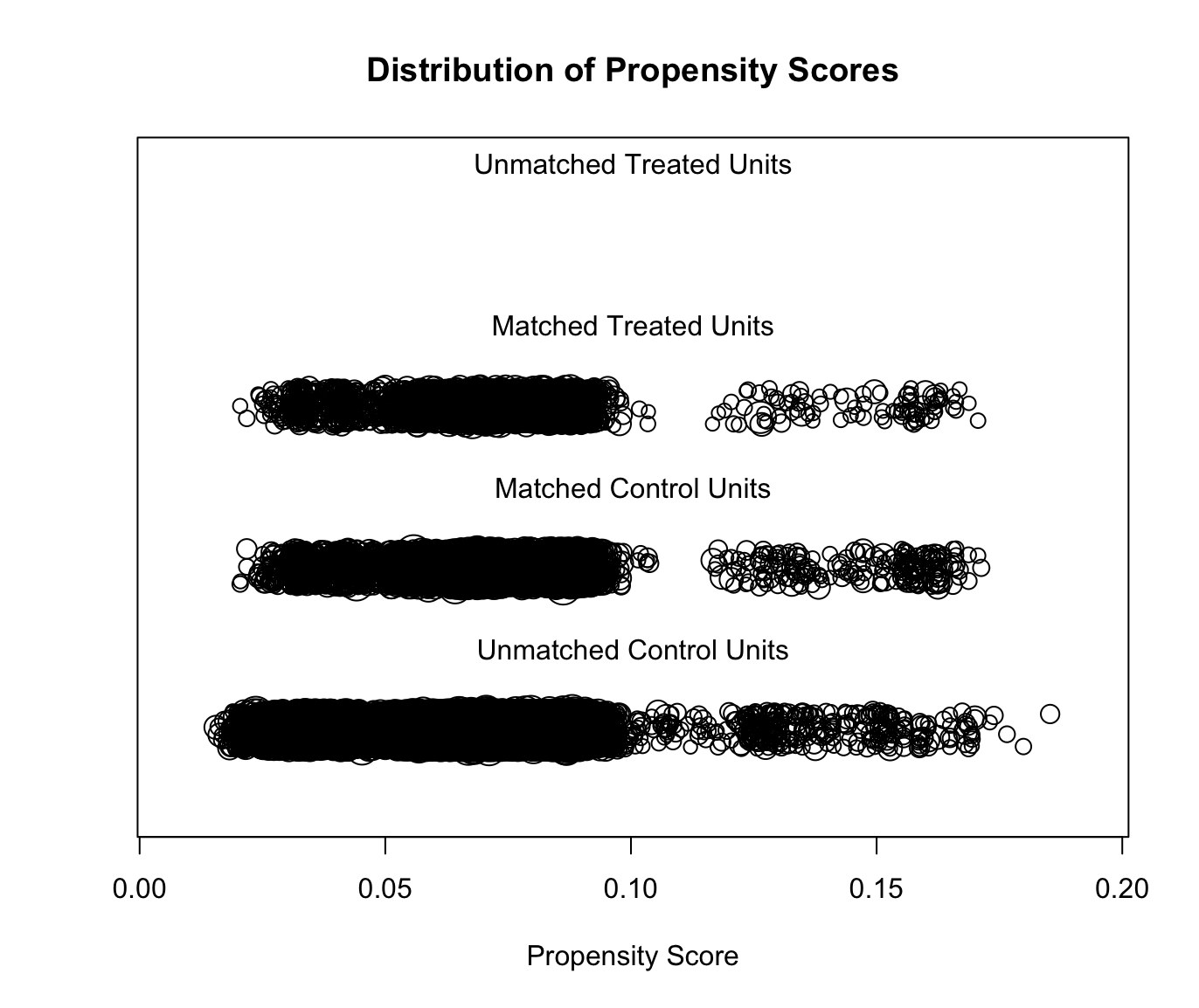

Supplement: Supplementary Table and Figure [file mmc1.docx]
